# Atypical presacral masses in Currarino syndrome: a case series

**DOI:** 10.1007/s00381-026-07486-4

**Published:** 2026-10-13

**Authors:** Ravi Ameet Patel, Rebecca Du, Erin Rowell, Julia E. Grabowski, Pouya Jamshidi, Nitin R. Wadhwani, Farres Obeidin, Jared T. Ahrendsen, Jean-Paul Wolinsky, Robin Bowman

**Affiliations:** 1 Department of Neurological Surgery, Northwestern University Feinberg School of Medicine, Chicago, IL 60611 USA; 2 Department of Surgery, Northwestern University Feinberg School of Medicine, Chicago, IL USA; 3 Division of Pediatric Surgery, Ann and Robert H. Lurie Children’s Hospital, Chicago, IL USA; 4 Department of Pathology, Northwestern University Feinberg School of Medicine, Chicago, IL USA; 5 Department of Pathology and Laboratory Medicine, Ann and Robert H. Lurie Children’s Hospital, Northwestern University Feinberg School of Medicine, Chicago, IL USA; 6 Department of Pathology and Laboratory Medicine, University of California Los Angeles, Los Angeles, CA USA; 7 Division of Pediatric Neurosurgery, Ann and Robert H. Lurie Children’s Hospital, Chicago, IL USA

**Keywords:** Currarino syndrome, Presacral mass, Atypical presentation, Case series

## Abstract

**Introduction:**

Currarino syndrome is a rare congenital disorder classically defined by the triad of sacral anomaly, anorectal malformation, and a presacral mass. While anterior meningoceles and teratomas are the most commonly reported lesions, an expanding body of literature suggests a broader spectrum of presacral pathology. We present three cases from our institution that expand the phenotypic range of presacral masses associated with Currarino syndrome, including lesions not previously well described in this population.

**Case presentation:**

Three cases are presented. Final pathologic diagnoses included myxopapillary ependymoma, mature cystic teratoma, and neuroendocrine tumor arising within a teratoma.

**Conclusion:**

These cases highlight the considerable heterogeneity of presacral lesions in Currarino syndrome and identify two underrecognized or previously unreported tumor types: myxopapillary ependymoma and neuroendocrine tumor. Surgical management must be individualized and often requires a multidisciplinary approach. Future studies will further clarify genotype–phenotype relationships and improve diagnostic and management strategies in this complex syndrome.

**Established facts:**

- Currarino syndrome is a rare congenital disorder classically defined by the triad of sacral anomaly, anorectal malformation, and a presacral mass.
- Presacral masses are most commonly anterior meningoceles and teratomas.
- A wider spectrum of lesions has been reported, underscoring the complexity of the syndrome and the importance of thorough evaluation and management of presacral pathology.

**Novel insights:**

- In Case 1, we report a previously undescribed association of myxopapillary ependymoma with a retrorectal teratoma in the setting of Currarino syndrome.
- Case 2 illustrates the potential for significant enlargement of presacral meningoceles with associated teratomas with a rare instance of prenatal diagnosis by ultrasound.
- Case 3 expands the spectrum of presacral pathology in Currarino syndrome by demonstrating a neuroendocrine tumor.

## Introduction

Currarino syndrome is a rare congenital disorder characterized by a triad of sacral anomaly, anorectal malformation, and presacral mass [1, 2]. First described by Guido Currarino in 1981, this syndrome is most commonly associated with mutations in the Motor Neuron and Pancreas Homeobox (MNX1) gene, resulting in abnormal development of the caudal notochord and hindgut during embryogenesis. Clinical presentation is variable, ranging from asymptomatic findings to significant neurologic, gastrointestinal, and urologic dysfunction.

Presacral masses are a defining feature of Currarino syndrome and are most commonly anterior meningoceles and teratomas [3, 4]. However, a wider spectrum of lesions has been reported, including enteric cysts, dermoid and epidermoid cysts, lipomas, leiomyosarcomas, yolk sac tumors, pelvic hamartomas, carcinoid tumors, and retrorectal cystic hamartomas. This diversity underscores the complexity of the syndrome and the importance of thorough evaluation and management of presacral pathology.

In this study, we present three cases of Currarino syndrome with presacral masses identified at our institution. These include a myxopapillary ependymoma, a markedly enlarged anterior myelomeningocele identified during pregnancy, and a neuroendocrine tumor arising within a teratoma. The aim of this case series is to further expand the recognized spectrum of presacral pathology associated with Currarino syndrome and highlight important considerations for diagnosis and surgical management.

## Case series

### Case 1: myxopapillary ependymoma with retrorectal hamartoma

#### Presentation and initial work-up

**Fig. 1:**
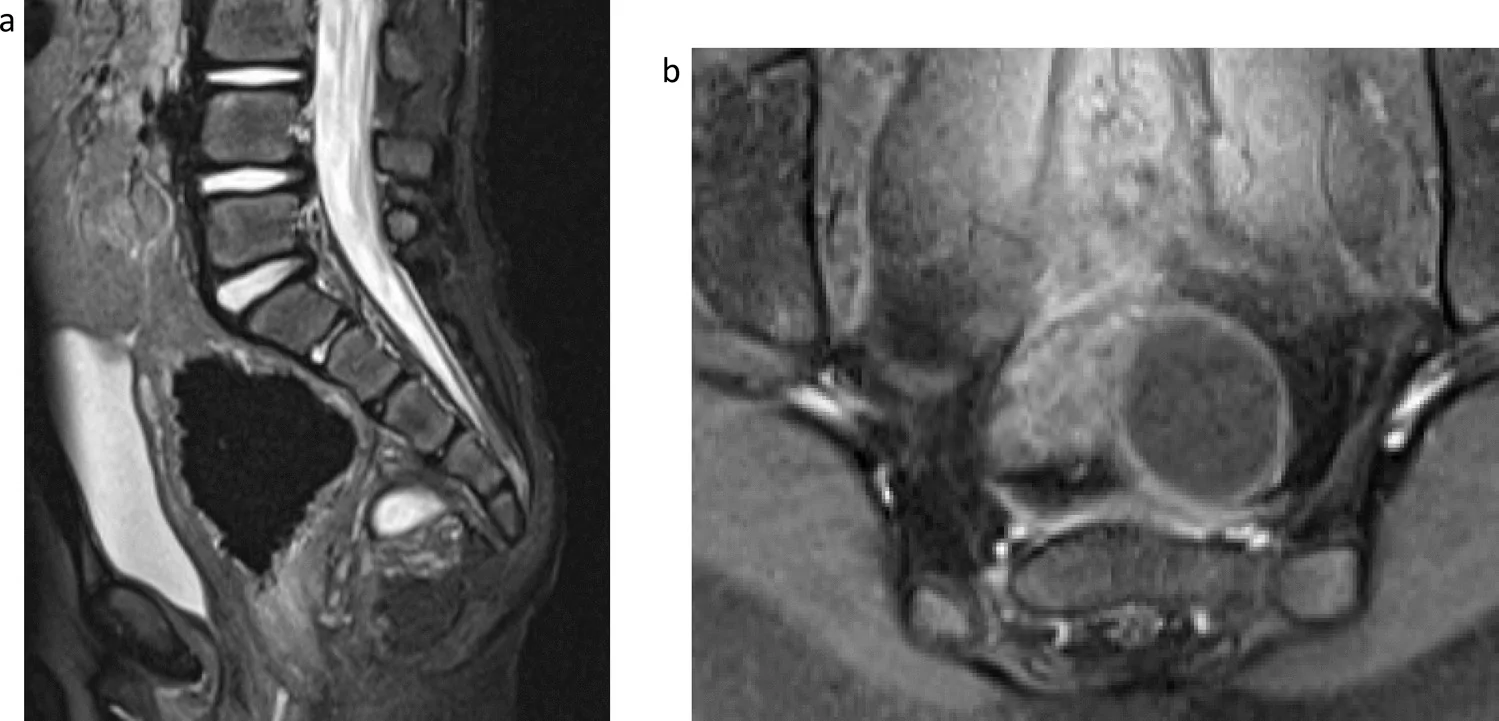
Case 1 Preoperative MRI: **a** sagittal T2-weighted and **b** axial T1-weighted post-contrast sequences demonstrating presacral mass with cystic, fat, and enhancing soft tissue components

A 34-month-old female with a twin brother diagnosed with Currarino syndrome presented for evaluation. She had no history of anorectal malformation but underwent screening magnetic resonance imaging (MRI) due to her family history, which revealed a tethered cord with a thin meningocele sac communicating with a presacral mass extending inferiorly beyond the coccyx. The mass appeared well-contained and demonstrated mixed cystic, fatty, and enhancing soft tissue components (shown in Fig. 1).

A comprehensive tethered cord evaluation was performed, including manual muscle testing (MMT) with physical therapy (PT), cystometrogram (CMG), and renal bladder ultrasound (RBUS). Baseline MMT demonstrated mildly decreased left toe flexors compared with right. CMG showed safe urodynamic pressures, increased bladder capacity for age, and low post-void residuals. RBUS demonstrated no hydronephrosis.

Following multidisciplinary evaluation with orthopedic surgery, urology, pediatric general surgery, and neurosurgery, treatment options were discussed with the family. The decision was made to proceed with surgical untethering, closure of the anterior meningocele, and resection of the presacral mass.

#### Operation

With the patient prone, a 5.9-cm (cm) incision was made from the upper sacrum to below the coccyx. Soft tissue and fascia were opened, revealing a bifid sacrum and the distal thecal sac, which descended through the sacral aperture into a presacral tumor. Utilizing a microscope, the dura was opened and the last exiting nerve roots were identified via intra-operative electromyograph (EMG) stimulation. The thickened filum was cauterized and transected to untether the spinal cord.

The dural attachment to the presacral tumor was circumferentially ligated distal to the last exiting nerve roots and transected from the distal thecal sac. The dura was closed in a watertight fashion. Following partial sacrectomy, the presacral mass was isolated from surrounding tissues and excised by the pediatric surgeons for pathological analysis. After ensuring bleeding was controlled, a drain was inserted into the presacral space, and the surgical site was closed in layers.

#### Pathology

**Fig. 2:**
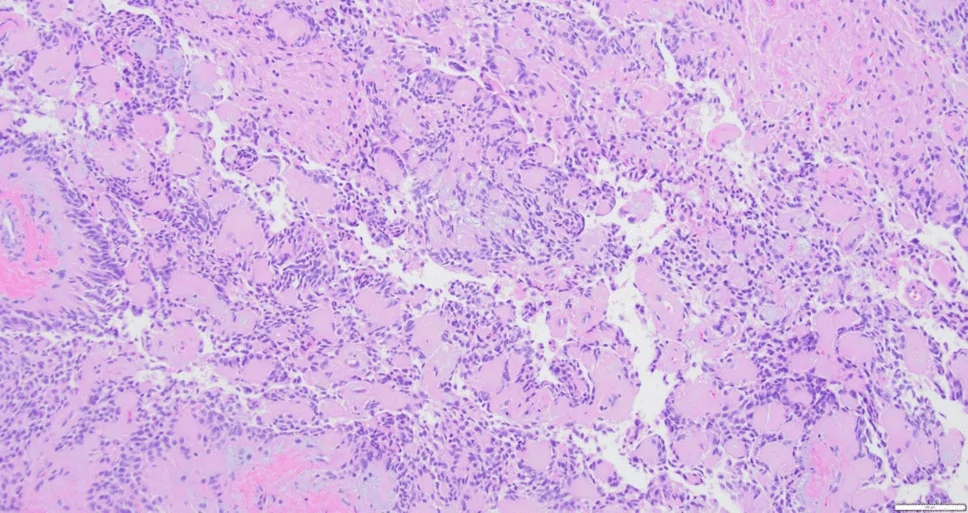
Case 1: H&E stain × 10 magnification tissue sample demonstrating WHO Grade 2 myxopapillary ependymoma

Gross examination of the tumor revealed two distinct solid components. Hematoxylin and Eosin (H&E) sections from one of the solid components revealed that the mass demonstrated a radial arrangement of cuboidal to epithelioid glial tumor cells around hyalinized fibrovascular cores forming a papillary configuration (shown in Fig. 2). There was extensive basophilic myxoid material around blood vessels and microcyst formation. Histologically this was diagnosed as a myxopapillary ependymoma. The second component recapitulated typical presacral elements commonly observed in retrorectal hamartomas. The latter component contained benign squamous lined cysts filled with keratinaceous material. There were also islands of mature glial tissue, mature adipocytes, fibrous tissue, and focal calcifications.

#### Post-operative course

Postoperatively, the patient did well. She was kept on flat bedrest 48 h with subsequent slow mobilization at home. At 4-month post-operative follow-up, the incision was well healed, and she was meeting all milestones. Imaging showed expected post-surgical changes with no evidence of residual or recurrent presacral mass.

### Case 2: giant anterior meningocele with a teratoma in 27-year-old female, diagnosed during prenatal ultrasound

#### Presentation and initial work-up

**Fig. 3:**
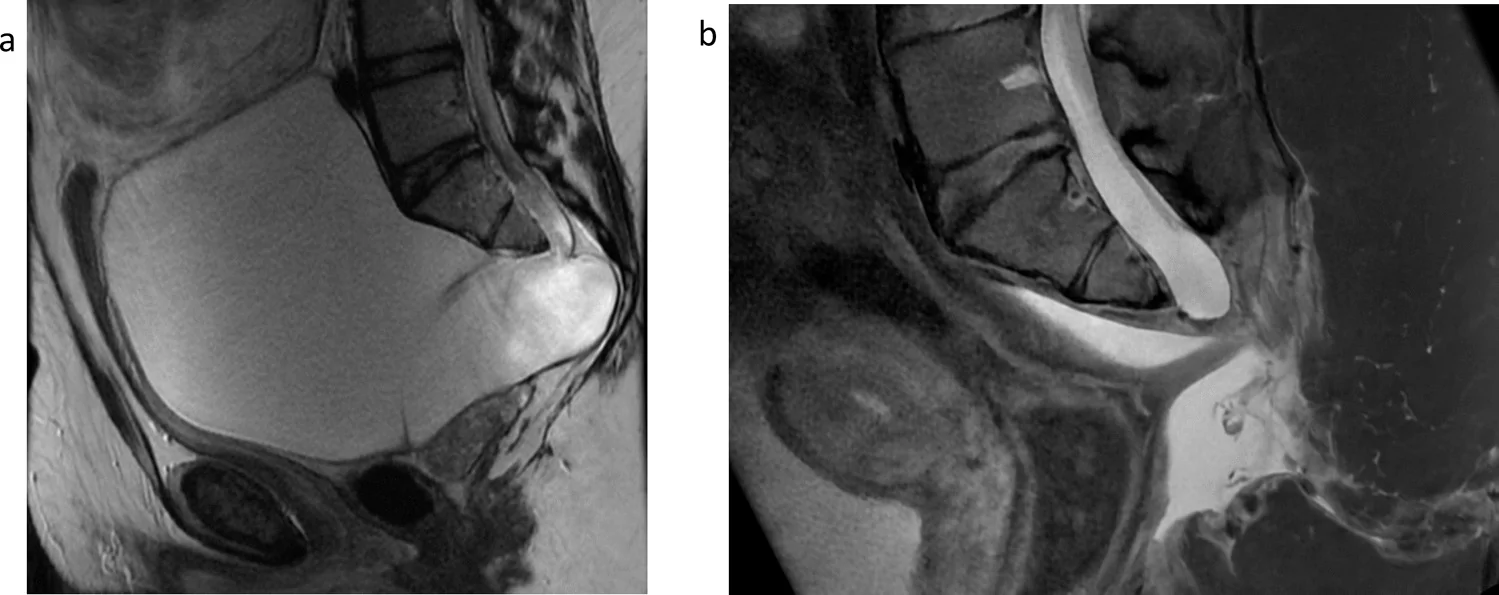
Case 2: **a** preoperative sagittal T2-weighted MRI of pelvis demonstrating giant anterior meningocele and teratoma; **b** post-operative sagittal T2-weighted MRI of pelvis demonstrating total resection of teratoma and closure of anterior meningocele

A 27-year-old G1P0 female presented at 28 6/7 weeks’ gestational age after MRI demonstrated a tethered spinal cord and a giant anterior meningocele measuring 11 × 18 × 13 cm (transverse × anteroposterior × craniocaudal), extending superiorly above the umbilicus, with an associated presacral mass concerning for a teratoma (42 × 50 × 20 mm) (shown in Fig. 3). The large pelvic mass was initially identified on a prenatal ultrasound.

At presentation, she reported a longstanding history of constipation requiring daily laxative use, urinary urgency with incontinence that worsened during pregnancy, and chronic headaches that improved with recumbency. Given concern for a teratoma, surgical resection in conjunction with general surgery, along with spinal cord detethering, was recommended following delivery and confirmation of a normal alpha-fetal protein (AFP) level. RBUS showed no significant abnormalities.

#### Operation

After placing a lumbar drain, the patient was positioned prone. A transverse incision at S2 was extended to expose the sacral fascia. The incision was opened midline with monopolar cautery and carefully dissected laterally. The paraspinal musculature over S1–S2 was dissected laterally. Inferiorly, the dorsal aspect of the pseudomeningocele was identified. Bilateral S1 and S2 laminectomies were completed, allowing extradural identification of the bilateral S2–S4 nerve roots.

A midline incision was created in the distal thecal sac, exposing a thickened filum descending into the anterior meningocele. Using microscopic dissection, the filum was freed from the surrounding nerve roots. Intraoperative EMG stimulation showed no functional activity, and the filum was subsequently cauterized and transected.

An intraoperative O-arm/computed tomography (CT) scan was obtained with a dissector placed within a dorsal sacral foramen to confirm bilateral exposure of the S2–S4 nerve roots.

Attention was then turned to the meningocele, which was opened and dissected to expose the teratoma. The lesion was carefully mobilized from the rectum and resected en bloc. The dura and meningocele were closed to prevent communication with the ventral presacral space, and the wound was closed in a standard multilayer fashion.

#### Pathology

**Fig. 4:**
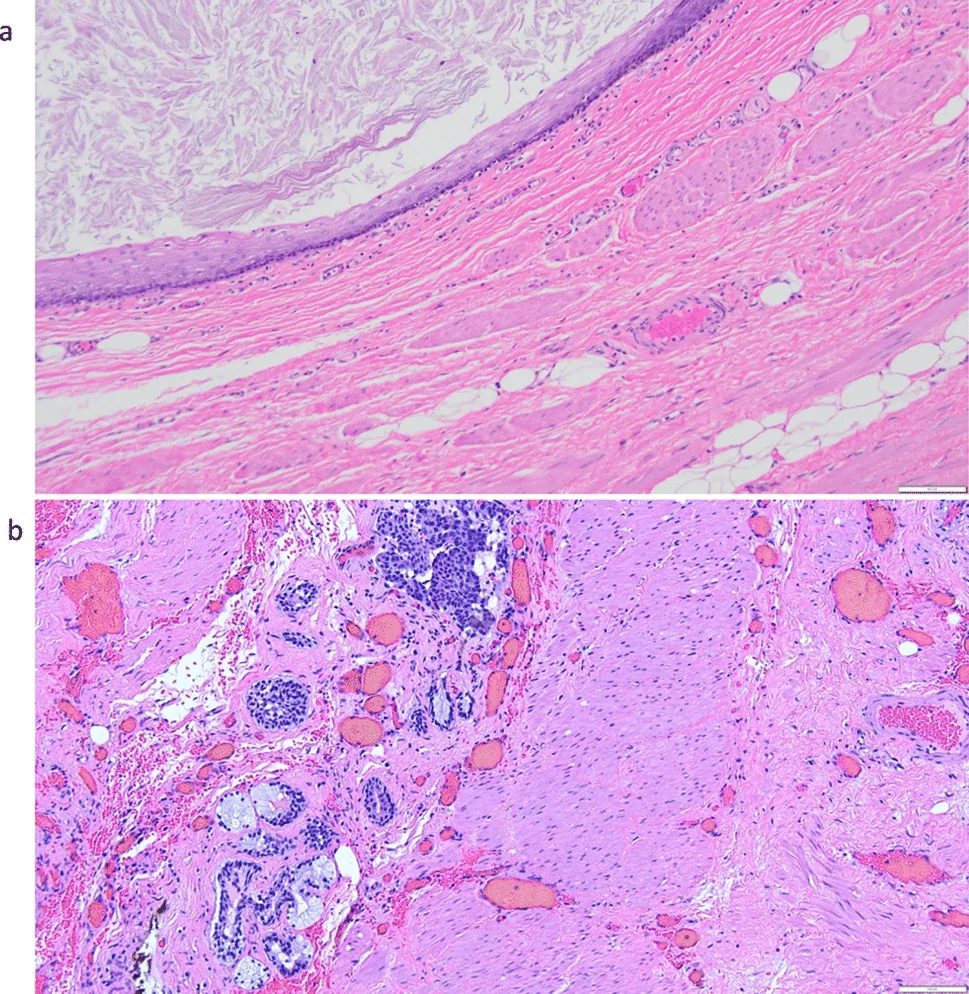
Case 2: **a** H&E stain × 10 magnification tissue sample demonstrating benign cystic lesion with squamous lining and acellular keratin debris; **b** H&E × 10 demonstrating benign glandular elements and muscle

Pathology was consistent with a mature cystic teratoma. Histologic sections showed a benign cystic lesion with squamous lining and acellular keratin debris. Benign glandular elements and muscle were also present (shown in Fig. 4).

#### Post-operative course

Postoperatively the patient was neurologically intact with full strength, preserved reflexes, and normal sensation. Cerebrospinal fluid drainage was initially maintained at 100 ml per hour (mL/h) via lumbar drain and reduced to 5 mL/h on post-operative day (POD) 5 due to spinal headaches. She remained at flat bedrest until POD 4, after which she was gradually mobilized with physical and occupational therapy. The lumbar drain was removed on POD 11, and she was discharged on POD 12.

At 3-month follow-up, her incision was well healed. MRI demonstrated gross total resection of the teratoma without evidence for recurrence and improvement in the presacral fluid collection. Post-operative urinary retention requiring intermittent catheterization resolved by the 3-month follow-up.

### Case 3: neuroendocrine tumor, grade 2, arising in a teratoma

#### Presentation and initial work-up

**Fig. 5:**
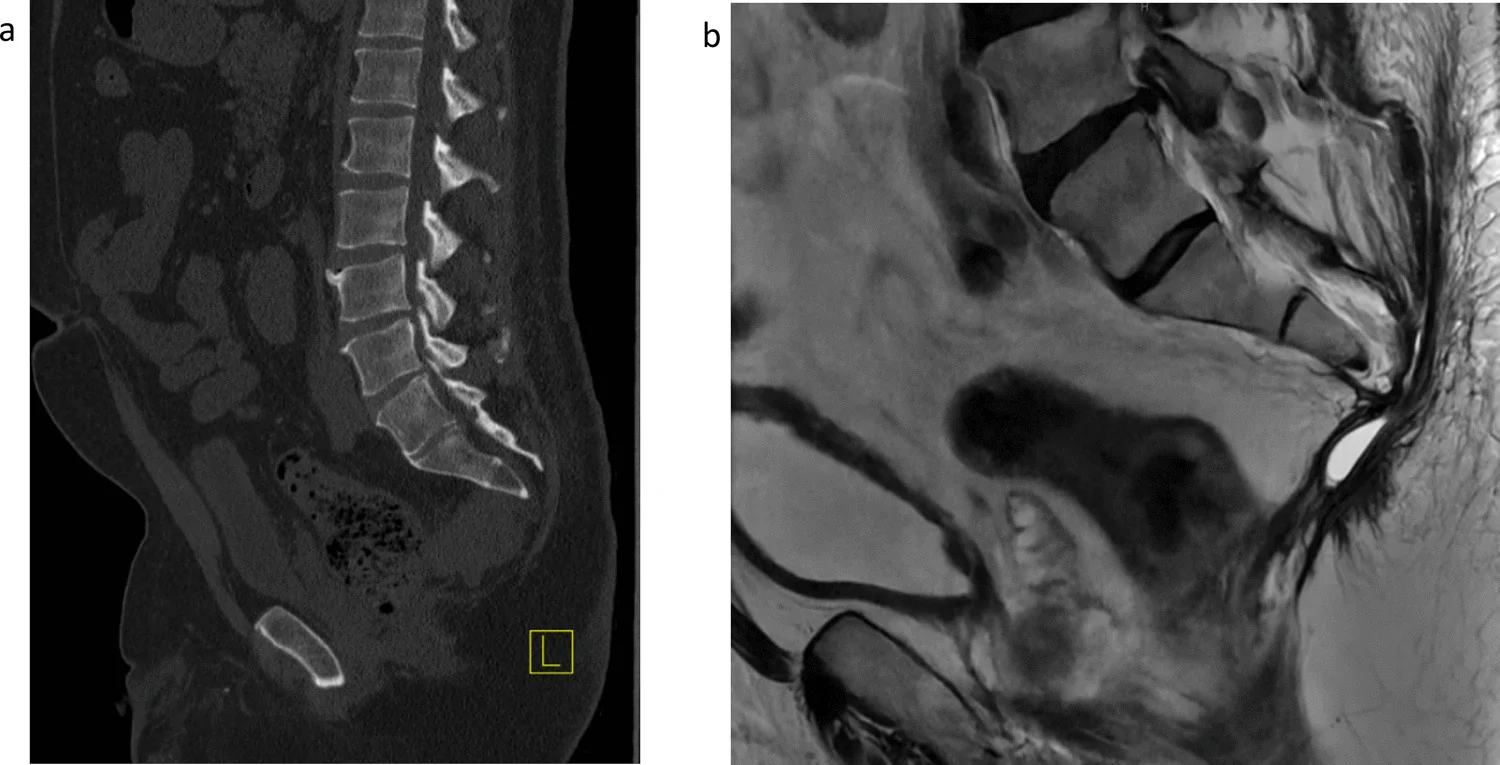
Case 3: **a** preoperative non-contrast CT abdomen-pelvis demonstrating presacral mass and anterior meningocele; **b** post-operative sagittal T2-weighted MRI of pelvis demonstrating gross total resection of presacral neuroendocrine tumor

A 53-year-old man presented with chronic back pain and was found to have sacral agenesis, a tethered cord, an anterior meningocele, and an associated presacral mass (shown in Fig. 5). His family history was significant for a daughter with Currarino syndrome who had previously undergone a tethered cord release, closure of anterior meningocele, and resection of presacral teratoma.

Urodynamic testing demonstrated normal bladder compliance and capacity, without evidence of uninhibited contractions or vesicoureteral reflux. Neurologic examination revealed mild weakness in right ankle dorsiflexion (5-/5), with otherwise preserved strength.

Given these findings, surgical management was recommended, and the patient elected to proceed with low sacral amputation, spinal cord detethering, closure of anterior meningocele, and en bloc resection of tumor.

#### Operation

With the patient in prone, a midline incision was made from S3 down to the coccyx through the subcutaneous tissue and fascia. The posterior aspect of S3 and sacral hiatus were exposed, and intraoperative O-arm/CT scan was obtained to localize the S4 foramina.

Following localization, a high-speed diamond burr was used to perform a right-sided osteotomy extending from the dorsal S4 foramen to the sciatic notch. The ventral periosteum was resected to expose the S4 nerve root along its course. The distal thecal sac, filum terminale, and the S5 and coccygeal nerve roots within the sacral hiatus were then identified. The thecal sac was opened, and the intradural filum was isolated using microscopic dissection. Following confirmation of absent neural function with EMG stimulation, the filum was coagulated and transected, thereby releasing the spinal cord. The dura was then closed primarily.

Attention was then directed to the distal sacrum. The S5 and coccygeal nerve roots were ligated. An osteotomy was created through the S4 vertebral body, rostral to the tumor, which was then connected to the lateral osteotomies to complete the low sacral amputation. Hemostasis of the middle sacral vessels was achieved with bipolar cautery.

Subsequently, the posterior muscle attachments posterior distal to S4 were dissected and divided bilaterally, including the piriformis muscle and the sacrospinous and sacrotuberous ligaments. In coordination with the general surgery team, a plane was developed between the tumor and rectum. Continued caudal dissection allowed progressive dorsal mobilization of the specimen, facilitating division of the anococcygeal ligament and en bloc resection of the tumor.

#### Pathology

**Fig. 6:**
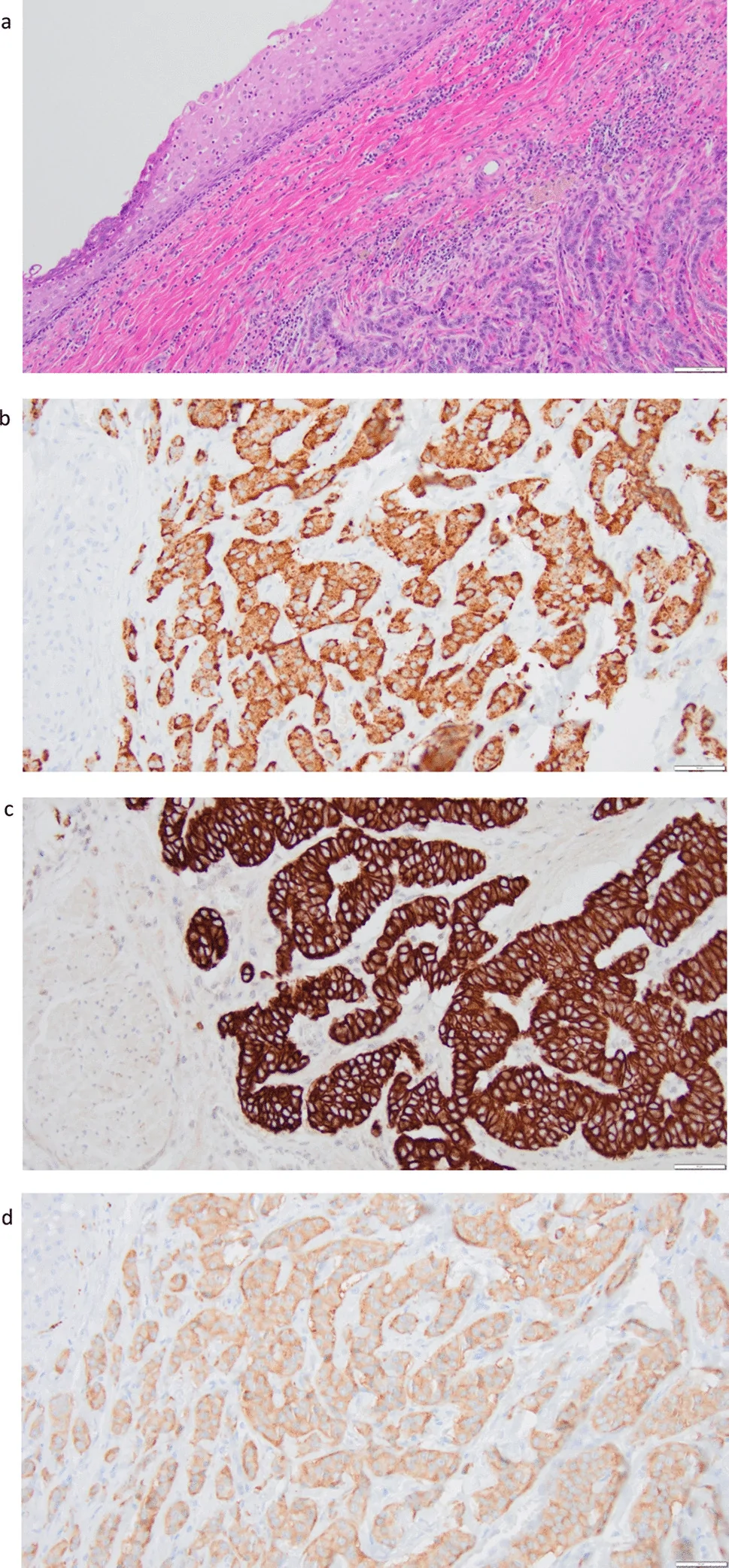
Case 3: **a** H&E stain × 10 magnification tissue sample demonstrating neuroendocrine tumor in teratoma; **b** chromogranin stain × 20 demonstrating neuroendocrine tissue; **c** cytokeratin AE 1/3 stain × 20 demonstrating epithelial tissue; **d** synaptophysin stain × 20 demonstrating neural tissue

Pathology was consistent with neuroendocrine tumor, World Health Organization (WHO) grade 2, arising in a teratoma. All surgical margins were negative for malignancy. There were areas of endodermal and ectodermal differentiation, which confirmed the background presence of teratoma. The bulk of the tumor was composed of a neuroendocrine neoplasm with variable differentiation, up to grade 2 (Ki-67: ~ 5%). The neuroendocrine tumor component showed extensive perineural invasion and focal lymphatic invasion (shown in Fig. 6).

#### Post-operative course

The patient was monitored in the intensive care unit postoperatively and transferred to the floor POD 1. After flat bedrest until POD 4, he was mobilized with therapy. A post-operative seroma identified on POD 7 was managed with percutaneous drainage. He was discharged in stable condition on POD 10.

At 6 months, the incision was well healed with no bowel, bladder, or sexual dysfunction. Neurological exam was normal. Imaging, tumor markers, and positron emission tomography (PET) scan demonstrated no evidence of residual, recurrent, or metastatic disease.

## Discussion

Currarino syndrome is a rare congenital disorder characterized by a triad of anorectal malformation, sacral anomaly, and presacral mass, with significant heterogeneity in clinical presentation and underlying pathology. This case series highlights the broad spectrum of presacral lesions encountered in Currarino syndrome and underscores both diagnostic and management considerations.

In Case 1, we report a previously undescribed association of myxopapillary ependymoma with a retrorectal teratoma in the setting of Currarino syndrome. While this entity has been briefly referenced in a pathology resource [5], to our knowledge, this represents the first formal case report of such a lesion in this population. Of note, it is not certain that these two pathologies are genetically related, as extradural, sacral myxopapillary ependymomas are rare, but they do occur as the filum extends beyond the thecal sac, to the tip of the coccyx. It is possible that this incidence of Currarino syndrome and myxopapillary ependymoma occurred coincidentally, and the underlying genetics needs further investigation.

Case 2 illustrates the potential for significant enlargement of presacral meningoceles with associated teratomas, despite minimal clinical symptoms at presentation. Notably, this case represents a rare instance of prenatal diagnosis by ultrasound.

Case 3 expands the spectrum of presacral pathology in Currarino syndrome by demonstrating a neuroendocrine tumor. Prior reports have described similar findings. Sable et al. reported an extragonadal mature cystic teratoma containing neuroendocrine tumor of intermediate malignant potential, ultimately classified as a WHO grade 2 neuroendocrine tumor based on elevated mitotic activity [6]. Rebelo et al. described a well differentiated neuroendocrine tumor characterized histologically by nests of polygonal cells with eosinophilic granular cytoplasm and “salt and pepper” chromatin, with immunoreactivity for CD56 and synaptophysin but negative for chromogranin [4].

Taken together, these surgical findings emphasize the marked heterogeneity of presacral pathology in Currarino syndrome, including the potential for neoplastic transformation or coexistence of multiple tumor types. These findings support surgical resection of presacral lesions not only for symptom relief but also for definitive diagnosis and mitigation of oncologic risk.

The optimal surgical approach in Currarino syndrome remains variable and is influenced by both patient anatomy and multidisciplinary team preference. In this case series, a single posterior approach allowed for simultaneous spinal cord detethering, closure of the anterior meningocele, and en bloc tumor resection. Alternate strategies have been described [7–9], including combined anterior–posterior approaches to facilitate mobilization and resection of large or complex lesions, as well as incorporation of posterior sagittal anorectoplasty in cases with associated anorectal malformations. These variations highlight the need for individualized operative planning.

Future directions include further delineation of genotype–phenotype correlations in Currarino syndrome, particularly in relation to presacral mass characteristics. In 1998, the homeobox gene, HLXB9, was identified as the major locus associated with dominantly inherited sacral agenesis [10]. These mutations were thought to impair wild-type function or represent loss-of-function alleles, with haploinsufficiency proposed as the underlying mechanism, particularly in patients with deletions of the 7q35-qter region [10]. Subsequent studies demonstrated that the majority of patients with Currarino syndrome harbor autosomal dominant mutations in HLXB9, which encodes the nuclear protein HB9 [3]. This gene was later renamed MNX1 and is located on chromosome 7q36 [11].

Despite this, MNX1 mutation analysis identifies pathogenic variants in only 57.4% of patients with Currarino syndrome, with no mutation detected in 7.7% of the familial cases and up to 68% of sporadic cases, suggesting that additional genetic alterations likely contribute to the development of this condition [12]. Advances in genomic technologies, including whole-exome sequencing and molecular karyotyping through copy number variation analysis, may help identify additional candidate genes and clarify the molecular mechanisms underlying Currarino syndrome [12]. Such work may ultimately improve understanding of the relationship between genetic variation and the diversity of presacral lesions observed in this disorder.

This study has several limitations. The small sample size reflects the rarity of Currarino syndrome and limits generalizability. As a single-institution case series, there is potential for selection and reporting bias. The heterogeneity of presentations and pathologies precludes definitive conclusions regarding optimal management strategies or causal relationships between Currarino syndrome and specific tumor types. In addition, incomplete genetic data limits the ability to draw robust genotype–phenotype correlations, and the relatively short follow-up period restricts assessment of long-term outcomes, including recurrence and functional sequelae.

## Data Availability

No datasets were generated or analysed during the current study.
